# Life expectations in early adolescence and the timing of first sex and marriage: evidence from a longitudinal survey in Ethiopia

**DOI:** 10.1186/s12978-021-01239-z

**Published:** 2022-06-13

**Authors:** David P. Lindstrom, Ida Sahlu, Tefera Belachew, Mulusew Gerbaba

**Affiliations:** 1 Population Studies and Training Center, Brown University, Providence, USA; 2 Department of Population and Family Health, Jimma University, Jimma, Ethiopia

**Keywords:** First sexual intercourse, First marriage, Schooling, Ethiopia

## Abstract

**Background:**

Raising the median age at first sexual intercourse and first marriage among females is a policy goal of the Ethiopian government. Education figures prominently in the government’s plans for achieving its goals, including primary and secondary schools; higher education; and out-of-school interventions such as youth centers, peer clubs, and youth associations In this study, we tested whether adolescents and youth who had high educational and occupational expectations at younger ages were at a lower risk of first sexual intercourse and marriage during adolescence and early adulthood.

**Methods:**

Data came from multiple waves of a longitudinal survey of households and adolescents conducted in southwestern Ethiopia. A measure of career expectations was created from educational and occupational expectations measured at baseline when the adolescents were ages 13–17. The occurrence and timing of first sexual intercourse (called first sex) and marriage were measured four years later in a wave 3 survey. Discrete-time logistic hazard regression models were applied to a person-year file to predict first sex for males and females separately and first marriage for females.

**Results:**

Male and female adolescents who had high career expectations at young ages were at a significantly lower risk of first sex during adolescence and early adulthood. Unlike the delaying effect of being in school, the effect of high career expectations did not wear off as adolescents aged. Among female adolescents, delaying first sex, staying in school, and having parents who desired them to marry at older ages were all associated with a significantly lower risk of marriage during adolescence and early adulthood.

**Conclusions:**

The educational and occupational expectations and family plans that youth develop early in adolescence influence the timing of the transition into sexual activity and marriage. Ethiopian youth who develop high career expectations delay first sex, which for female youth is a key predictor of age at first marriage. Adolescents’ perceptions of parents’ expectations for them are strongly associated with their own expectations and behavior.

## Plain Language summary

Raising the median age of their first heterosexual intercourse, or first sex, and marriage for females is a policy goal of the Ethiopian government. Research in Africa has found evidence of a positive association between higher levels of completed schooling and older ages at first sex and marriage among females. A substantial body of research conducted in the United States shows that youth who develop high educational and occupational expectations at young ages also tend to delay first sex and marriage. High expectations motivate youth to stay in school longer and avoid behaviors, such as early sex and marriage, that put their goals at risk. Evidence of a similar association in Ethiopia, however, has been lacking because of the scarcity of longitudinal data. In this study, we used data from the Jimma Longitudinal Family Survey of Youth conducted in southwestern Ethiopia to examine the impact of early career expectations and marriage plans on the risk of first sex during adolescence among male and female youth and on the risk of first marriage during adolescence among females. Using discrete-time logistic hazard regression models, we found that adolescents who had high career expectations at ages 13–17 were significantly less likely to have their first sexual intercourse over the following four years. We also found that the protective effects of being in school on delaying first sex wore off with time, but the effect of high career expectations persisted. Finally, we found that parents’ desire for an older age at marriage for their daughters had a significant delaying effect on daughter’s marriage.

## Background

Ethiopia is a young nation with close to 40% of the estimated population of 115 million between the ages of 10 and 24 [1]. Although women’s age at marriage has risen among recent cohorts, it remains relatively low. Among women ages 25–49, the median age at first marriage is 17.1 years and the median age at first heterosexual intercourse, or first sex, is 16.6 years [2]. The recent National Adolescent and Youth Health Strategy 2016–2020 of the Ethiopian government identifies raising the median age at first sex and first marriage among females to 18 years or above as one of its outcome targets [3]. Education—including primary and secondary schools; higher education; and out-of-school interventions such as youth centers, peer clubs, and youth associations—figures prominently in the government’s plans for achieving its goals. The emphasis on early-life interventions is in line with the government’s identification of the life-course approach as one of the guiding principles in its strategic framework [3]. The life-course approach emphasizes the important influence of early events, experiences, and contexts on choices, behaviors, and life chances at older ages. Most studies on age at first sex and first marriage use retrospective data from cross-sectional surveys that make it difficult to establish a causal relationship between early life experiences, expectations, and later behaviors. In this study, we use longitudinal survey data collected from a cohort of youth in southwestern Ethiopia to assess the influence of life expectations, experiences, and family context measured in early adolescence on the risk of first sex and first marriage during the adolescent and early adult years. We provide evidence that youth who had higher career expectations at young ages tended to delay first sex and marriage, even after taking into account the protective effects of being in school.

School is a highly influential institutional setting that shapes the life course of youth. A positive relationship between years of schooling and age at first sex and first marriage is well documented in the demographic literature [4–8]. Part of the effect of education on delaying sexual initiation and marriage results from being a student. The role incompatibility hypothesis argues that the sequencing of age-appropriate roles in the early life course is strongly regulated by social norms and institutional rules and regulations [9]. In most societies, schooling, especially at the primary and secondary levels, is viewed as preparing children and youth for adult roles and responsibilities. Being a student is often considered incompatible with being married and taking on marital and parental roles. Indeed, in many African countries, girls who become pregnant are required to leave school [10]. Keeping adolescents in school longer, however, is not always equally effective in delaying sexual initiation for male and female youth, especially at older adolescent ages. Meekers and Ahmed [11] for example, found in a study of urban youth in Botswana that being in school reduced the risk of being sexually active among female adolescents ages 13–18 but had no effect on males.

Schooling exposes youth, and especially girls, to an expanded range of life opportunities and choices as alternatives to early marriage and traditional gender roles [12]. Pursuing professional occupations and jobs in the modern sector of the economy requires higher levels of education and hence that youth remain in school at ages when marriage traditionally occurs. The opportunity cost perspective focuses on the incompatibility between meeting the time demands of attaining higher levels of schooling and early work experience and the demands of marriage and parenthood [13]. Implicit in the approach is the importance of aspirations as a motivation for staying in school. Youth with high educational and occupational aspirations recognize that early marriage reduces the chances of school progression and ultimately the attainment of higher status occupations.

Research on adolescents in the United States has explored the impact of early career and family plans on age at marriage. Vesely et al. [14] found that youth with higher future aspirations were less likely to have had first sex, and Lauritsen [15] found that high educational aspirations were associated with lower levels of sexual activity among White youth but not among Black youth. Hockaday et al. [16] found that adolescent girls with high educational expectations and life aspirations were at a lower risk of pregnancy. Vernon et al. [17] found that adolescent girls with lower expectations about future jobs were at a higher risk of early pregnancy. Other studies found that high educational expectations among female youth were associated with postponement of childbearing [16, 18], as were higher educational and occupational aspirations [19].

Research on the influence of early educational and occupational aspirations and expectations on the initiation of sexual activity and entry into marriage provides compelling evidence that youth who develop high aspirations and future life plans tend to transition into sexual activity and marriage at older ages. However, this research is based primarily on the U.S. experience. Very little is known about the influence of early career aspirations and expectations on age at first sex and marriage in the African context, where the expansion of educational opportunities beyond the primary level is relatively new and occupational opportunities in the modern sector are more limited. In addition to the constraints on educational and job opportunities that boys face, many girls face low expectations at home and gender discrimination at school and in the labor market, all of which raise doubts about the influence of early expectations on outcomes at older ages.

## Methods

### Study design

Our analysis uses multiple waves of data from the Jimma Longitudinal Family Survey of Youth (JLFSY) conducted in southwestern Ethiopia [20]. The JLFSY employed a multistage stratified, cluster sample design to randomly sample households from six neighborhoods in the city of Jimma (population 120,000), three nearby market towns, and nine rural areas adjacent to the towns. The neighborhoods in the city of Jimma were randomly selected with selection probabilities proportionate to size. The market towns were purposively selected to provide variation in economic structure. In the six neighborhoods in Jimma, a street-by-street enumeration of all households was conducted to construct a sampling frame. In the three towns and rural communities, household registration lists maintained by the local authorities were used as sampling frames after a random spot check confirmed the completeness of the lists.

A baseline household survey was completed in 2005–2006 with 3695 households. Up to one boy and one girl ages 13–17 were selected from each of the sampled households for participation in multiple waves of an adolescent survey. In households with more than one eligible adolescent of the same gender, a Kish table was used to randomly select one of them. Sampled youth were approximately uniformly distributed from ages 13 to 15, with the relative percentages dropping at ages 16 and 17 as youth left the household for marriage or migration. A baseline adolescent survey was completed several months after the household survey with 2084 adolescents. Interviewers were recruited from the study area and were required to have a minimum of 12 years of completed schooling and be fluent in the two dominant local languages, Amharic and Afan Oromo, and in English. Versions of the survey questionnaires were printed in Amharic and Afan Oromo. The adolescent interviews were conducted by an interviewer of the same gender as the adolescent and in a private space inside or near the adolescent’s place of residence. Second and third wave adolescent surveys were conducted in 2006–2007 and 2009–2010. The response rates for the baseline household and adolescent surveys were close to 100%, with very low refusal rates (see Tables 1, 2). In the second and third adolescent survey waves, multiple visits were made to households in an attempt to locate youth who moved away from the study area on return visits. The refusal rate rose to 2% in the second and third wave adolescent surveys, and the nonresponse rate rose from 6% in the second wave to 25% in the third wave. The overall response rates of 92% in the second wave survey and 73% in the third wave survey are quite good for a longitudinal study of adolescents. The primary reasons for lost to follow-up were that youth migrated to another place in Ethiopia (64%), migrated to another country (32%), or left to attend university (4%).

**Table 1:** Response rates for baseline household and wave 1–3 adolescent surveys, JLFSY, 2005–2010

	Household survey	Adolescent surveys		
	Wave 1 2005–2006	Wave 1 2005–2006 ages 13–17	Wave 2 2006–2007 ages 14–18	Wave 3 2009–2010 ages 17–21
Respondents	99.9%	98.9%	91.8%	72.7%
Nonrespondents		0.1%	6.2%	25.4%
Refusals	0.1%	1.0%	2.0%	1.9%
Total	100.0%	100.0%	100.0%	100.0%
Effective sample size	3700	2107	2104	2102

Effective sample size excludes subjects who were disabled or died

**Table 2:** Variable definitions, JLFSY 2005–2010

Dependent variables (wave 3)
First sex = 1 if first sex in a given life year and 0 otherwise
First marriage = 1 if first marriage in a given life year and 0 otherwise
Independent variables
Life expectations (wave 1, age 13–17)
Career expectations: Standard normal index based on factor analysis of highest expected years of schooling and expected occupation coded according to the Standard International Occupational Prestige Scale
Expected age at first marriage: Age at which respondents expected to marry
Personal autonomy: Standard normal index based on factor analysis of responses to four questions regarding ability to make life decisions: 1. Could you decide to have a job that your parents do not approve of? 2. Could you marry a person whom your parents did not approve of? 3. Do you think you will decide who your future spouse will be? 4. If your parents chose a partner for you whom you did not want to marry, would you tell them so? High values correspond to greater influence over decisions
Gender equality: Standard normal index based on factor analysis of responses to 10 statements on women and men’s roles: 1. A woman should always listen to her husband. 2. A husband should have the final say in all major family matters. 3. Marriage by abduction is acceptable. 4. There is nothing a woman can do if her husband has a mistress. 5. Female circumcision is a practice that should continue. 6. Normally a man should not have to do housework. 7. A woman could be mayor. 8. A wife should be allowed to request a divorce. 9. A women should be allowed to marry a man of her choice. 10. It is acceptable for females to buy condoms. In wave 1, the responses were agree or disagree. High values of the index correspond to more gender egalitarian attitudes
School participation
In school (time varying, waves 1, 2, and 3) = 1 if student in a given life year and 0 otherwise
Membership in youth clubs (wave 1, age 13–17) = 1 if member of a youth club and 0 otherwise
Family and community environment (wave 1, age 13–17)
Religiosity: Standard normal index based on factor analysis of responses to five questions on religious practices: 1. In the last year, on religious days in which you should attend church/mosque, how often did you go? 2. In the last year, on religious days of fasting, how often did you fast? 3. Do you or have you ever received religious instruction outside of your home, for example Koranic school or Bible classes? 4. How often do you pray? 5. How important is religion to you? High values of the index correspond to higher levels of religious observance
Parents’ desired age of daughter’s marriage: Average of the age at which the respondent thinks her father and her mother want her to marry
Parents’ highest year of schooling: Highest year of schooling completed by father or mother
Household wealth: Standard normal index based on factor analysis of ten household measures: owns radio, television, electric stove, bicycle, motorcycle, home; has electricity, protected source of drinking water, toilet, and non-dirt floor
Female headed household = 1 if female household head and 0 otherwise

### Measures

The second and third wave adolescent survey questionnaires asked youth whether they had ever had sexual intercourse and the age at which they first had intercourse. To reduce the prevalence of underreporting of premarital sexual activity the interviewers used nonverbal response cards for soliciting responses to sensitive questions about sexual knowledge, attitudes, and practices. The cards were designed to address concerns about social desirability bias and privacy and were field tested in a survey of youth in an area adjacent to the JLFSY study area [21, 22]. All three adolescent survey waves also asked marital status and age at first marriage.

The focus of our analysis is the impact of life expectations developed at younger ages on the risk of first sex and first marriage during the adolescent ages. Our measures of life expectations and the family environment come from the first wave survey when the adolescents were ages 13–17. Our measure of career expectations is a standard normal index based on a factor analysis of highest expected year of schooling and expected occupation coded with the Standard International Occupational Prestige Scale [23].^1^ The use of a composite index based on educational and occupational expectations to measure career expectations follows Rojewski and Yang [26] and evidence from prior studies of the close interrelatedness of the two concepts [27–29]. We expected high career expectations to be associated with delayed first sex among both male and female youth. Prior studies provide strong evidence of a close connection between the initiation of sexual activity and entry into marriage in Ethiopia [6, 30, 31], especially for females. In our analysis of first marriage, we used as a predictor the adolescent’s expected age at first marriage. Bayer [32], in a study of U.S. young adults, found that their expected age of marriage measured at ages 17–18 was the single best predictor of their ages at marriage. We also included a measure of personal autonomy that is a standard normal index based on a factor analysis of responses to four questions regarding perceived ability to make life decisions regarding occupation and partner selection without parents’ approval. High values on the index correspond to high levels of autonomy. We expect high levels of autonomy among male youth to be associated with earlier age at first sex because it is likely to correlate with less parental control. The expected effect of autonomy for females is ambiguous because less parental control could result in both more opportunities to engage in sexual activity and a greater desire to challenge traditional gender roles and pursue higher education and a career. We included a gender equality index based on a factor analysis of responses to 10 statements on women and men’s roles. High values on the index correspond to more gender egalitarian attitudes. Plotnick [33] found that U.S. female youth who supported more egalitarian views of women’s roles were more likely to be sexually active than female youth with more traditional views. Others have found that male-dominant gender role attitudes among male youth are associated with greater risk taking [5]. Research on U.S. female youth has found that less traditional gender attitudes are associated with delayed first birth [19]. We expect in the Ethiopian context that more egalitarian gender roles will be associated with delayed first sex among both male and female youth. Our expectations for female youth are the opposite of findings among U.S. youth because of the close relationship in Ethiopia between the timing of first sex and first marriage, and our expectation that female youth with more egalitarian attitudes will want to avoid early sexual activity if it places them at a greater risk of early marriage. Similarly, we expect that female youth with more egalitarian gender attitudes will delay first marriage.

We measured school participation with two variables: a time-varying indicator of in school status and an indicator of participation in a youth club. Youth clubs in the study area include sports, academic, cultural activities, civics, reproductive health, and vocational training. Studies of youth in the United States found that participation in after school sports was associated with later age at first sex among females [34, 35], and earlier age at first sex among males. Studies also found that participation in sports and other school-based extracurricular activities was associated with higher educational aspirations [36] and other positive developmental outcomes [37, 38]. In the Ethiopian context we expect participation in youth clubs to be associated with delayed first sex among male and female youth and delayed marriage among female youth because of the expected positive association between participation in clubs and higher educational and occupational aspirations.

To measure the early family environment, we included a standard normal religiosity index that is based on a factor analysis of five questions on religious practices, training and importance. Crockett et al. [39] in a study of U.S. youth found that higher levels of religiosity were associated with older age at first sex. They suggested that greater religious involvement reflected personal beliefs regarding sexual conduct, and that time spent in religious activities may have reduced the opportunities for youth to engage in sexual activities. We expect high levels of religiosity to be associated with delayed first sex among males but earlier first sex and first marriage among females. Because of the close connection between sexual initiation and marriage among females in Ethiopia, and the strong connection between traditional beliefs and early marriage, we expect highly religious females to transition into first sex and first marriage at earlier ages than less religious females. For males, sexual initiation is not so closely linked to marriage and therefore we expect higher levels of religiosity to be associated with delayed first sex. The baseline adolescent questionnaire asked the adolescents the age at which they believed their father and their mother wanted them to get married. We took the mean of these two responses to measure the adolescents’ perception of parents’ desired age at marriage. We expect female youth who believed their parents wanted them to delay marriage to marry at older ages. We included parents’ highest year of schooling and an index of household wealth to control for socioeconomic status, and we included an indicator variable for female headed household. Axinn and Thornton [40] found in the United States that higher values of parents’ education and mothers’ ideal age for children to marry were associated with older age at marriage. Multiple studies in the United States have also found that living in a single parent household was associated with lower age at first sex [39, 41–43], earlier age at first birth [19], and earlier age at marriage [44]. Finally, we included control variables for the level of urbanization in the study sites.

### Models

To estimate the effects of early expectations and family environment on age at first sex and first marriage, we used discrete-time logistic hazard regression models. Because of the low prevalence of first marriage among adolescent males in the study area, we modeled the hazard for first marriage only for females. We began exposure to the risk of first sex and first marriage at age 11. We constructed a person-year data file that followed the youth from age 11 up to the age of the event or right censoring. Duration times to first sex and first marriage were right censored at the time of the wave 3 adolescent survey if the youth had not had first sex or first marriage by the time of the interview. The analysis file includes data for 870 males and 651 females who completed at least the first and third wave surveys. The vast majority of these cases also completed the second wave survey as well. At the time of the third wave survey the youth were ages 17–21. We used sample weights for generating descriptive statistics and in our regression models, and we estimated the regression models with robust standard errors that adjusted for clustering at the community level.

### Descriptive statistics

Sample attrition is a common concern in longitudinal studies. Sample attrition can introduce bias into study results to the extent that subjects who are lost to follow-up are different from subjects who remain under observation. Table 3 presents the sample means for the dependent and independent variables in our analysis for the male and female youth who remained in the sample and for the lost to follow-up youth. By the time of the wave 3 survey, 17% of the in-sample males had first sexual intercourse, as had 24.6% of the in-sample female youth. Less than 1% of the in-sample male youth and 11.3% of the in-sample female youth were married by the time of the wave 3 survey. As a point of comparison, we found the percentages of 17- to 21-year-old females and males in urban areas of Ethiopia that had sexual intercourse and had ever been married in the 2011 Ethiopia Demographic and Health Survey [45]. Close to 23% of male youth had first sex and 2.8% had ever been married. Among females, 32% had first sex and 23.1% had ever been married. Although the two samples are not directly comparable, the figures from the DHS suggest that the JLFSY sample may be biased downward with respect to ever married females.

**Table 3:** Sample means for males and females by in-sample and lost to follow-up status, JLFSY 2005–2010

	Males		Females	
	In- sample	Lost to follow-up	In- sample	Lost to follow-up
First sex	0.171		0.246	
First marriage	0.008		0.113	
Life expectations (age 13–17)				
Career expectations	0.066	0.214*	0.122	− 0.043***
Expected age at marriage			25.9	26.0
Personal autonomy	0.036	0.206*	0.211	0.104*
Gender equality	− 0.036	− 0.077	0.383	0.311
School participation				
Membership in youth clubs (age 13–17)	0.600	0.633	0.574	0.534
Family and community environment (age13–17)				
Religiosity	− 0.131	− 0.242	0.108	0.176
Parents’ desired age of daughter’s marriage	27.5	26.8	25.0	24.6*
Parents’ highest year of schooling	4.277	4.889*	5.114	4.911
Household wealth	0.225	0.292	0.406	0.353
Female headed house	0.188	0.242	0.235	0.241
City	0.680	0.680	0.718	0.740
Town	0.084	0.078	0.081	0.075
Number of observations	870	162	651	349

Significance levels for difference of means/proportions test, sample weights applied

*P < 0.10, **P < 0.05, ***P < 0.01

Among males, the lost to follow-up youth tended have slightly higher career expectations, slightly higher levels of personal autonomy, and more educated parents. The differences on these three measures are marginally significant at the 0.10 level. None of the differences on the other nine measures listed in Table 3 are statistically significant. In the case of females, the lost to follow-up youth tended to have lower career expectations, lower levels of personal autonomy, and lower parents’ desired age at marriage. Only the difference in career expectations is statistically significant at the 0.01 level, whereas the other two differences are marginally significant at the 0.10 level. As was the case with males, none of the differences on the other nine measures are statistically significant. These results along with the information on reasons participants are lost to follow-up suggest that some of the males who left the study area were likely pursuing better educational and occupation opportunities, whereas the selective out-migration of females with lower career expectations suggests other processes were at work. Based on other information collected in the field, we believe the two primary reasons females left the study area were for domestic work in the capital city and international locations (mainly the Gulf states) and for marriage.

To explore the issue of selective sample attrition further, we estimated a logistic regression model using baseline sample characteristics to predict participants lost to follow-up (Table 4). The model provides an additional test of whether youth lost to follow-up were significantly different from the in-sample youth on observed characteristics that are also related to the timing of first sex and first marriage. Among males, parents’ highest year of schooling is the only variable that predicted sample attrition at the 0.05 significance level. Males who were lost to follow-up tended to have parents with higher levels of education. None of the other individual or family-level variables are significant. In the case of females, career expectations is the only statistically significant variable at the 0.05 level of significance. Females with low career expectations tended to be at a higher risk of leaving the study area. Parents’ desired age at marriage is marginally significant at the 0.10 level: females whose parents wanted them to marry at young ages tended to be at a higher risk of being lost to follow-up. If our hypotheses about the negative effects of career expectations and parents’ desired age at marriage are correct, then these results are consistent with higher than expected sample attrition of females who were at a higher risk of early marriage.

**Table 4:** Parameter estimates from logistic regression models predicting lost to follow-up, males and females, JLFSY 2005–2010

	Males	Females
Life expectations (age 13–17)		
Career expectations	0.169	− 0.200**
Expected age at marriage		0.028
Personal autonomy	0.118	− 0.100
Gender equality	− 0.102	− 0.087
School participation		
Membership in youth clubs (age 13–17)	0.044	− 0.031
Family and community environment (age 13–17)		
Religiosity	− 0.070	0.118
Parents’ desired age of daughter’s marriage		− 0.040*
Parents’ highest year of schooling	0.037**	− 0.003
Household wealth	0.033	− 0.067
Female headed house	0.354	− 0.060
City	− 0.437	0.515
Town	− 0.451*	0.324
Rural (ref.)		
Number of observations	1032	1000
Pseudo *R*^2^	0.016	0.016

Robust standard errors adjusted for clustering at the community level, sample weights applied

*P < 0.10, **P < 0.05, ***P < 0.01

## Results

We turn now to the results from the discrete-time logistic hazard regression models predicting first sexual intercourse and first marriage. In the first sex models and the first marriage model, we tested for interactions between age (time-varying) and in school status. The interactions tested whether the predicted negative effect of being in school on the hazard of first sex and first marriage wore off with age. We found significant age and in school interactions in the first sex models but not in the first marriage model. We report in Table 5 the results from the male and female first sex models with the interaction term and the results for the first marriage model without the interaction term. Among male youth, high career expectations were associated with a significantly lower hazard of first sex. Being in school also significantly lowered the hazard, but the significant positive effects of age and the age–in school interaction indicate that the protective effect of in school status wore off as youth got older. Parents’ highest year of schooling, female headed household, and living in an urban area were all associated with a significantly higher hazard of first sex. Parents with higher levels of education may have been more permissive than other parents with their sons and provided them with greater freedoms. We suspect male youth in female headed households tended to have less parental supervision than youth in two-parent households. Youth in urban areas also have more opportunities than youth in rural communities to avoid the supervision of parents, all of which place them at a higher risk of first sex. The basic pattern of results for female youth was very similar to that of male youth. Higher career expectations and being in school at younger ages were associated with a lower hazard of first sex, and living in an urban area was associated with a higher risk. In comparison to males, the magnitude of the effect of career expectations for females was more than twice as large. In contrast to males, parents’ education and living in a female headed household had no effect on the hazard of first sex for females. This last result is consistent with the greater supervision and more restricted freedom of movement that unmarried girls experience in Ethiopia [6].

**Table 5:** Parameter estimates from discrete-time hazard models predicting first sexual intercourse and first marriage, JLFSY 2005–2010

	First sex males	First sex females	First marriage females
Had first sex in a prior year			2.764***
Life expectations (age 13–17)			
Career expectations	− 0.143**	− 0.363***	
Expected age at marriage			− 0.049
Personal autonomy	0.165	− 0.067	− 0.288
Gender equality	0.136	0.014	0.210
School participation			
In school (time varying)	− 5.002**	− 3.517**	− 0.681*
Membership in youth clubs (age 13–17)	0.092	0.025	0.139
Family and community environment (age13–17)			
Religiosity	− 0.001	0.036	0.157
Parents’ desired age of daughter’s marriage			− 0.078**
Parents’ highest year of schooling	0.043***	− 0.025	− 0.033
Household wealth	− 0.002	− 0.216	0.042
Female headed house	0.610**	− 0.301	− 0.588**
City	1.639***	1.115**	− 0.581
Town	1.210**	0.790*	0.006
Rural (ref.)			
Duration dependence			
Age (time varying)	0.302***	0.276***	0.260***
Age × in school interaction	0.275**	0.196***	
Number of life years	7339	5314	5504
Number of observations	870	651	651
Pseudo *R*^2^	0.208	0.139	0.282

Robust standard errors adjusted for clustering at the community level, sample weights applied

*P < 0.10, **P < 0.05, ***P < 0.01

In summary, consistent with our predictions career expectations formed at younger ages influenced the timing of first sex at older ages. Not only did being in school at younger ages lower the hazard of early sexual initiation, higher educational and occupational expectations lowered the hazard as well. In addition to testing for an interaction between age and in school status, we also tested for an interaction between career expectations and age to test whether the delaying effect of high career expectations on age at first sex also wore off with time. We found no evidence of interaction effects in the male and female models. Unlike the effect of being a student, which changed from placing youth at a lower hazard of first sex to eventually a higher hazard of first sex, high career expectations were consistently protective of early sexual initiation throughout the adolescent years.

**Fig. 1:**
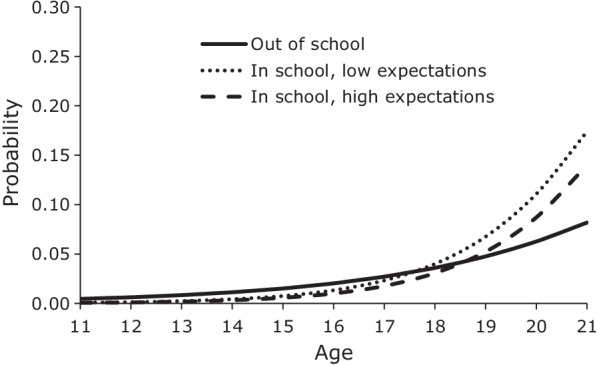
Mean predicted probability of first sex, males by student status and career expectations, JLFSY 2005–2010

**Fig. 2:**
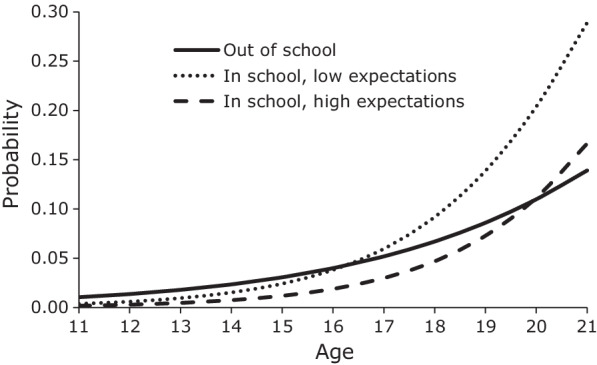
Mean predicted probability of first sex, females by student status and career expectations, JLFSY 2005–2010

We graphed the predicted probabilities from the discrete-time logistic hazard regression models to show the relative impact of in-school status and adolescents’ career expectations on the risk of fist sex. Figure 1 presents the mean age-specific probabilities of first sex for males if they are out of school and if they are in school with low career expectations or high career expectations. Low expectations have a value of − 1 (1 standard deviation below the mean) and high expectations have a value of 1 (1 standard deviation above the mean). All other covariates are as observed. The graph shows that up to around ages 17–18 males who were in school had a lower probability of first sex than males who were out of school. However, after ages 17–18, the probability of first sex for males who were still in school rapidly surpassed that of out-of-school males. Having high career expectations prolonged the protective effects of student status for males, but only modestly. In the case of females, the delaying effect of high career expectations was much larger than for males, and it extended by more than one year the age at which the probability of first sex for females in school was below that of out-of-school females (Fig. 2).

Our results provide clear evidence that high career expectations are associated with delayed first sex for adolescents in Ethiopia. These results are consistent with a large body of research from other countries that connects aspirations in early adolescence to a wide range of later life outcomes, including educational [29] and occupational attainment [27, 46], as well as the initiation of sexual experience [14]. Theory and evidence, however, also suggest that family background has potentially significant moderating effects on the influence of early life aspirations and expectations on subsequent behavior [47, 48]. Youth expectations and behavior are influenced not only by individual traits but also by family context, which provides the resources needed to realize life plans and aspirations [49]. Family cultural and financial resources play a critical role in academic achievement and can either reinforce youth aspirations and expectations or dampen them [26, 27]. In accord with our findings and the research literature, we pursued our analysis of age at first sex one step further and tested for the moderating effects of family cultural and financial capital, measured by parents’ highest year of schooling and household wealth. Parents with higher levels of education understand the role and value of education in future occupational attainment and are more aware of behaviors that lead to educational success than parents with little or no school experience. They also serve as models for their children. We reestimated the first sex models for males and females with interactions between career expectations and parents’ schooling and career expectations and household wealth added separately. The parents’ education interaction was statistically significant in both the male and female samples, and the wealth interaction was significant only in the female sample.

**Fig. 3:**
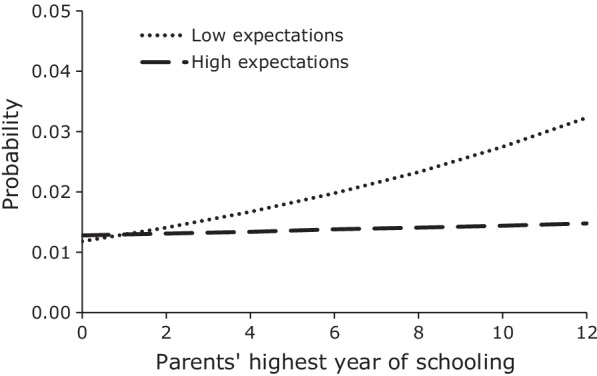
Mean predicted probability of first sex, males by career expectations and parents’ education, JLFSY 2005–2010

**Fig. 4:**
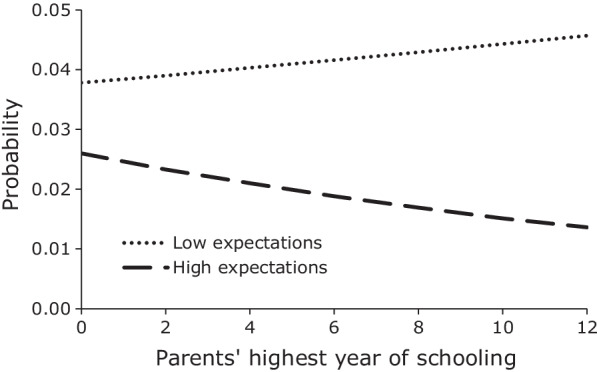
Mean predicted probability of first sex, females by career expectations and parents’ education, JLFSY 2005–2010

**Fig. 5:**
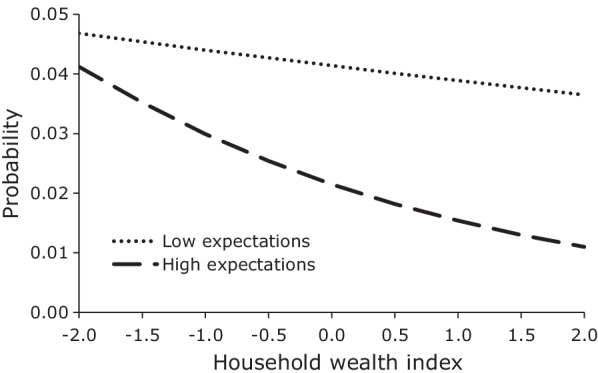
Mean predicted probability of first sex, females by career expectations and household wealth, JLFSY 2005–2010

Figures 3, 4 graph the mean predicted probabilities of first sex among male and female youth for low and high career expectations across the range of parents’ highest year of schooling, and Fig. 5 graphs the mean predicted probabilities of first sex among female youth by low and high career expectations for different values of the household wealth index. In the case of males, the interaction model reveals that the positive relationship between parents’ schooling and the hazard of first sex only existed among youth with low career expectations. Among male youth with high career expectations, parents’ education had no apparent effect. The result is still consistent with our earlier conjecture that more educated parents provide greater freedom to their sons than less educated parents do, but it suggests that either this greater freedom is only given to sons who demonstrate low educational and occupational expectations or that it is only taken advantage of by such sons. Regardless, the result from the interaction model provides clear evidence of the significant effect of high career expectations on lowering the hazard of early sexual initiation. In the case of female youth, the relative effect of career expectations also increases with increases in parents’ education. However, in contrast to male youth, the delaying effect of high career expectations becomes increasingly large with increases in parents’ education. Interestingly, a slight positive slope in the probability of first sex for female youth with low career expectations suggests that the leniency or reduced supervision that we observed among male youth with low career expectations and more educated parents also seems to apply to female youth with low career expectations. Figure 5 graphs the mean predicted probabilities of first sex among female youth with low and high career expectations by level of household wealth. As was the case with parents’ education, the relative effect of career expectations increases with increases in household wealth. However, in contrast to parents’ education, the probability of first sex declines with increases in household wealth for all levels of career expectations, but it declines the most for females with high career expectations.

We now turn to the hazard of first marriage among female youth. The final column in Table 5 presents the results from the model predicting the hazard of first marriage among female youth. Because of the very close relationship in Ethiopia between the timing of first sex and first marriage, we included in the model a time-varying indicator variable of ever had first sex lagged by one year. We also added to the model expected age at first marriage and the parents’ desired age at first marriage. We did not include career expectations in the model of first marriage because it was correlated with ever had first sex and expected age at marriage. Having had first sex in a prior year increased the hazard that a female youth married by a factor of close to 16 (e^2.764^). In contrast, being in school was associated with a significantly lower hazard of marriage. Unlike with age at first sex, we found no evidence that the protective effect of being a student wore off during adolescence for the risk of marriage. Contrary to our predictions, we found no evidence that expected age at marriage had any effect on the risk of marriage during adolescence. However, we did find that female youth’s perception of parents’ desired age at marriage did have a very significant effect. Female youth who believed their parents wanted them to marry at older ages were at a lower risk of early marriage than other female youth. We also found that female youth in female headed households were at a significantly lower risk of early marriage. This finding stands in stark contrast to first sex among male youth in similar households. One possible explanation for the delaying effect of female headship on marriage is that female household heads needed the labor and/or earnings of an adolescent daughter and therefore had an interest in delaying their marriage.

**Fig. 6:**
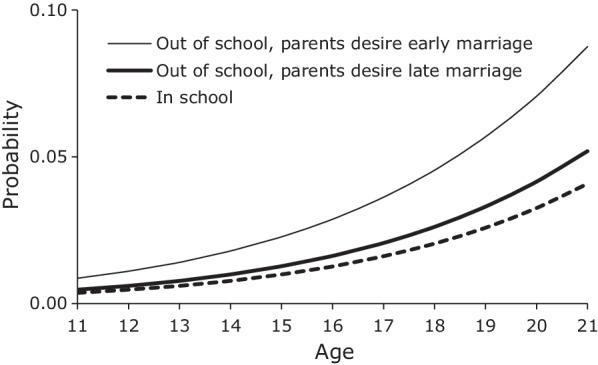
Mean predicted probability of first marriage, females by student status and parents’ desires, JLFSY 2005–2010

We present in Fig. 6 the mean predicted probabilities of first marriage among female youth by student status and perceived parental preferences regarding age at marriage. Being in school was associated with a lower probability of marriage for females at all ages, not just younger ages. The graph also highlights the comparatively large effect that parents’ desires for age at marriage had on the timing of daughters’ marriage. Having parents who desired an older age at marriage (1 standard deviation above the mean) lowered the probability of marriage among out-of-school females close to the probability of marriage for in school youth. Having parents who desired a younger age at marriage (1 standard deviation below the mean) approximately doubled the probability of marriage compared to in school youth.

## Discussion

The influence of early adolescent career aspirations and expectations on later occupational outcomes is well documented [50, 51]. Research demonstrates that adolescent aspirations and expectations are relatively stable across the adolescent years [52, 53] and that earlier aspirations and expectations are strongly predictive of aspirations and expectations at older ages [26]. In this study, we used measures of educational and occupational expectations. Aspirations are defined in the literature as what youth ideally would like to do, and expectations are what they believe they realistically will attain [54–56]. Hence, expectations come closer to what youth believe is possible. Indeed, research finds that expectations tend to be lower than aspirations [51, 57, 58] and that over time aspirations move closer to expectations [54]. The premise of our study is that educational and occupational expectations will influence the timing of first sex and marriage to the extent that youth view early sex and early marriage as obstacles to achieving their career goals. We found strong evidence that male and female youth who had high career expectations in early adolescence were at a significantly lower risk of first sex during adolescence and early adulthood than were youth with low expectations. The delaying effect of high expectations operated in addition to the protective effect of student status. However, unlike student status, which wore off by late adolescence and eventually placed youth at an even higher risk of first sex, the delaying effect of high expectations persisted across the adolescent years. Youth in the study area who developed high educational and occupational expectations at young ages recognized that entering into sexual relationships could make it more difficult for them to achieve their career goals. The potential threat that early sex poses for high educational and occupational attainment is greater for females than males in Ethiopian society, and our results are consistent with this threat. The relative effects of high career expectations were substantially larger for female compared to male youth. Female youth who had career expectations 1 standard deviation above the mean had a hazard of first sex that was 30% (e^−0.363^) lower than that of females with average career expectations. Among males who had career expectations 1 standard deviation above the mean, their hazard of first sex was only 13% (e^−0.143^) lower than that of males with average career expectations.

Family context clearly plays an important role in the development of adolescents’ career expectations and life plans. Although we did not study these processes here, we did include measures of family resources and structure that influence adolescent opportunities to engage in early sex. In our main effects models, we found that male youth with more highly educated parents and in female headed households were at a greater risk of first sex. We interpreted both of these results as stemming from greater freedom and permissiveness and less parental supervision. Among female youth, both factors had negative effects on the risk of first sex, although neither coefficient was statistically significant, suggesting that in similar family contexts girls experienced less freedom and greater supervision than boys did. We also tested whether parents’ education and household wealth mediated the effects of career expectations on the risk of first sex. We found clear evidence that, indeed, the delaying effect of high career expectations was largest among male and female youth who had highly educated parents, as well as among female youth in wealthier households.

In addition to increasing the relative influence of high career expectations on delaying the timing of first sex, parents play an instrumental role in the development of children’s educational and occupational expectations. The strong positive association between parents’ and children’s aspirations and expectations is one of the most consistent findings in the research literature on adolescent development and achievement [27, 47, 59]. In our sample, the correlation coefficient for youth’s perception of their parents’ career expectations for them and their own career expectations was 0.71 for males and 0.68 for females.

Delaying the start of sexual activity is especially important for female adolescents who want to delay marriage. The connection between first sex and first marriage is very high in Ethiopia and has remained fairly stable over recent decades [6, 60]. In our sample, unmarried female youth who had sexual intercourse were at 16 times the risk of marriage in a subsequent year than female youth who had not had first sex. The comparatively large effect of first sex on marriage provides a strong incentive for female youth with high educational and occupational expectations to delay sexual activity. Once adolescent girls in Ethiopia become sexually active, the chance that they will soon marry increases greatly. As was the case with first sex during the early and mid-adolescent years, being a student prolonged the time to first marriage. However, in contrast to the case of first sex, we found no evidence of a decline in the protective effect of student status over time. Although being in school at upper levels of education may place young women at a higher risk of first sex, they are still at a lower risk of marriage than similar women who aged out of school.

Parents communicate to children their goals and expectations. We included in our model of marriage the age at which female youth believed their parents wanted them to marry. Parents’ preferred age at marriage had a larger and more significant impact on girls’ age at marriage than girls’ own expected age at marriage. This result is strong evidence of one of the ways that parents can influence their children’s behavior, by setting expectations for the timing of key transitions. We also found that girls in female headed households were at a lower risk of early marriage. Female headed households in Ethiopia are typically more economically disadvantaged and vulnerable than male headed households. We suggested that the need of a daughter’s labor and income assistance might be one explanation for this finding. It is also possible that mothers are more likely than fathers to prefer an older age at marriage for their daughters and that in female headed households, mothers have more influence on their daughter’s behavior than in male headed households.

Sample attrition is a shortcoming of the JLFSY data. We found that male youth who were lost to follow-up tended to have higher career expectations than males who remained in the sample and that female youth who were lost to follow-up tended to have lower career expectations than females who remained in the sample. Given the significant positive relationship we found between age at first sex and career expectations among males, the selective attrition of males with high career expectations would bias the estimated effects of career expectations upward if sexually active males tended to leave the study area. In the case of females, the selective attrition of females with low career expectations would bias our estimated effects of career expectations on first sex and first marriage downward if the transition into sexual activity and marriage was connected to young women’s movement out of the study area.

Our overall findings are consistent with findings from studies of adolescents and youth in the United States and in other countries. Our study is one of very few of African youth that uses longitudinal survey data to test the effects of early life expectations on the timing of first sex and marriage at older ages. Our results confirm that even in a development context where the expansion of public education is relatively recent and professional employment in the modern sectors of the economy is comparatively new, youth who develop life plans with high educational and occupational expectations recognize the risks that early sex and marriage place on their chances of achieving their goals. An important caveat to our findings is that decisions about the timing of first sex and first marriage are not always entirely under the control of adolescents. A survey of adolescents and young adults conducted in an area adjacent to the JLFSY study area estimated that approximately 8 percent of young people ages 13–24 who had ever had sex were raped at the time of first sex [22].

## Conclusions

Our results provide confirmation that in Ethiopia keeping male and female adolescents in school is protective of early sexual initiation, but only up to late adolescence. However, instilling high educational and occupational expectations in children at young ages can have long-term positive effects on encouraging them to delay sexual activity and marriage. The potential effects of high expectations are especially large for girls. Parents play a large role in both keeping their children in school longer and encouraging them to develop career goals and life plans. Parents’ goals and expectations are especially important for girls. In Ethiopia it is traditional for girls to marry during adolescence, so the age at which parents desire their daughters to marry has a significant impact on girls’ age at marriage, even more so than what girls themselves want. It is important that schools communicate to parents the lasting importance of instilling and supporting high expectations with respect to school and work in their children. Of course, not all children will be nor can be in the top professional ranks, but higher aspirational goals within any occupation that keep youth in school longer and delay sexual initiation and marriage will have positive effects not only on human capital formation but on a range of other subsequent life outcomes.

## Data Availability

The data from the JLFSY are available upon request to David_Lindstrom@brown.edu.

## Abbreviations

JLFSY: Jimma Longitudinal Family Survey of Youth
DHS: Demographic and Health Survey

## Footnotes

1 The Standard International Occupational Prestige Scale has not been validated in contemporary Ethiopia. The last two published efforts to develop occupational prestige rankings for Ethiopia were based on small samples of primary and secondary students interviewed in 1958 by Schack [24] and university students interviewed in the late 1960s by Brown [25]. Both studies found that students ranked government and occupations requiring higher education at the top and unskilled occupations at the bottom, and Brown found that the Ethiopian rankings were highly correlated with American rankings of comparable occupations.
